# Zoonotic *Baylisascaris procyonis* Infection in Raccoons, Mississippi, USA, 2023–2024

**DOI:** 10.3201/eid3110.250658

**Published:** 2025-10

**Authors:** Bryan L. Huerta-Beltrán, Huan Zhao, Stephen Mills, Joshua Berry, William Janous, Javian Ervin, Karleigh Butler, Aamani Kalluru, Fritz Valerio, Blake Stefano, Trent Selby, Nicole M. Phillips, Steven Everman, Graham T. Rosser, Charlotte V. Hobbs, Richard S. Bradbury, Scoty M. Hearst

**Affiliations:** The University of Southern Mississippi, Hattiesburg, Mississippi, USA (B.L. Huerta-Beltrán, N.M. Phillips); James Cook University, Townsville, Queensland, Australia (H. Zhao, R.S. Bradbury); Mississippi College, Clinton, Mississippi, USA (S. Mills, J. Berry, W. Janous, J. Ervin, K. Butler, A. Kalluru, F. Valerio, T. Selby, S.M. Hearst); Mississippi Department of Wildlife, Fisheries, and Parks, Jackson, Mississippi, USA (B. Stefano); The University of Mississippi Medical Center, Jackson (S. Everman); Mississippi State University, Mississippi State, Mississippi, USA (G.T. Rosser); University of Alabama at Birmingham, Birmingham, Alabama, USA (C.V. Hobbs)

**Keywords:** Baylisascaris procyonis, parasites, Mississippi, raccoons, zoonoses, infectious disease surveillance, United States

## Abstract

*Baylisascaris procyonis*, an emerging zoonotic parasite, causes clinically significant visceral, ophthalmologic, and neurologic disease in humans. We screened raccoons (n = 46) collected from central and southern Mississippi for *B. procyonis* by necropsy (13.0% prevalence) and droplet digital PCR of feces (26.7% prevalence). Further surveillance to determine raccoon infection rates throughout Mississippi is indicated.

*Baylisascaris procyonis* roundworms are emerging zoonotic parasites that cause clinically significant visceral, ophthalmologic, and neurologic disease in humans. The 3 major categories of human *Baylisascaris procyonis* infection are neural larva migrans, ocular larva migrans, and visceral larva migrans (*1*–*3*). Raccoons (*Procyon lotor*) are the definitive host for *B. procyonis* roundworms (*2*,*3*). Case reports in the literature describe life-threatening neurologic disease manifestations of eosinophilic meningitis, which causes substantial illness and death (*3*). North American raccoons are human-habituated and thrive in urban and suburban areas, posing a serious health risk to humans (*4*,*5*). 

Human *B. procyonis* infection occurs after ingestion of eggs from raccoon feces in contaminated soil from the environment, commonly in latrine areas in which raccoons defecate (*6*). Female *B. procyonis* roundworms produce >100,000 eggs/day/worm, and infected raccoons shed millions of eggs through fecal matter at latrine locations (*1*,*7*). Younger children and persons with developmental delays are more likely to become infected because of increased likelihood of ingesting eggs because of pica behaviors (*3*). In the United States, 33 cases of human *B. procyonis* infection have been reported (*3*). *B. procyonis* has previously been reported in states neighboring Mississippi, USA, in the southeast (*5*,*8*–*10*). We report the presence of *B. procyonis* roundworms in raccoons in Mississippi.

## The Study

We collected raccoons (n = 46) during 2023–2024 from 8 different counties in central and southern Mississippi (Hancock, Harrison, Hinds, Humphreys, Lincoln, Pearl River, Yazoo, and Warren Counties). We used dog-proof traps or live traps with a scientific collection permit issued by the Mississippi Department of Wildlife Fisheries and Parks (permit no. 0207241); some collections were made from donated hunter-harvested raccoons. Raccoons were stored at −20°C until examined by necropsy. During necropsy, we examined the raccoon’s small and large intestines for the presence of *B. procyonis* nematodes. We collected and stored *B. procyonis* adult and juvenile worms in 70% ethanol before DNA analysis. At the time of necropsy, 45 of the raccoons contained fecal matter; we collected fecal samples collected from the large intestines of those animals and stored at −20°C before DNA analysis (Appendix). All animal protocols used in this study were approved by the Mississippi College Institutional Animal Care and Use Committee (protocol no. 072723) and Institutional Review Board (protocol no. 111621).

**Figure 1:**
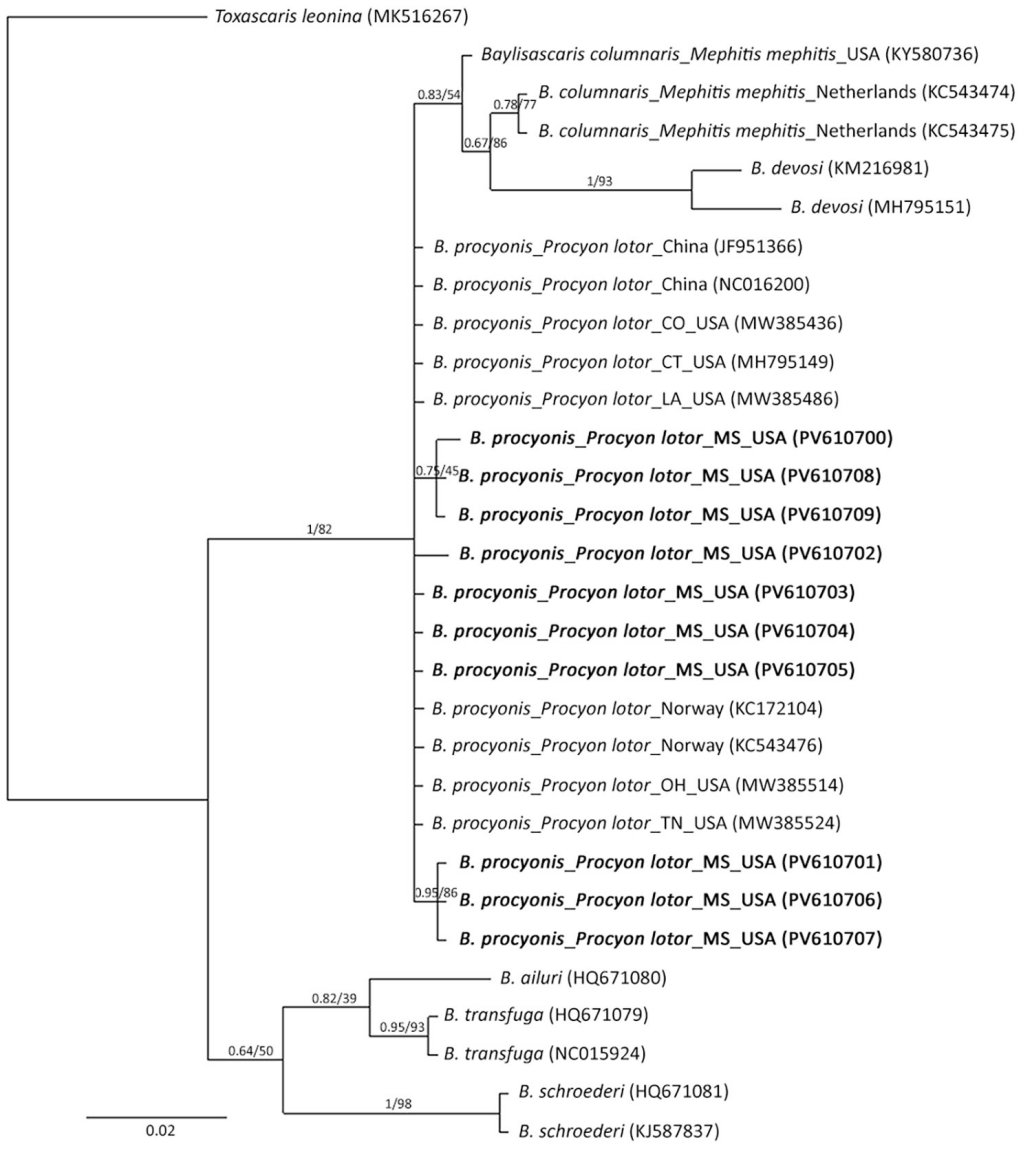
Phylogenetic trees of *Baylisascaris procyonis* *cox1* genes from nematodes (n = 10) collected by necropsy in raccoons, Mississippi, USA, 2023–2024. Analysis was conducted using the general time-reversible plus gamma model. Bayesian posterior probabilities and bootstrap support values (1,000 bootstrap replicates) are shown for each node. GenBank accession numbers are in parentheses. Scale bar indicates number of substitutions per site.

We confirmed *B. procyonis* presence in necropsied raccoons by morphology (Appendix Figure, panel A), Sanger sequencing and droplet digital PCR (ddPCR) analysis of fecal samples (n = 45), and sequencing of helminth DNA (n = 46) (Table; Appendix). PCR and Sanger sequencing using *B. procyonis cox1*, *cox2*, *ITS1*, *18s*, and *28S* primers revealed 100% identity to *B. procyonis* sequences in GenBank (accession nos. MW385486, AF179908, NC_016200, U94368, MZ092854) (*11*,*12*). We submitted DNA sequences to Genbank (accession nos. PV593814 [*18s*], PV594051 [*28s*], PV594407 [*ITS1*], PV600642 [*cox2*], and PV610700–9 [*cox1*]). Phylogenetic analysis of the *cox1* sequences (n = 10) showed 100% identity with *B. procyonis* nematodes collected from raccoons in the United States, China, and Norway (Figure 1) (*11*,*12*). Necropsy of raccoons revealed *B. procyonis* positivity in 2/8 counties (Humphreys and Yazoo); positivity rates were >50%. The number and length of *B. procyonis* nematodes found in infected adult and juvenile raccoons varied (Appendix Table 1). Higher infection rates were seen in juvenile raccoons than in adult raccoons, as previously reported (*1*). Identification of *B. procyonis* eggs in fecal samples was hindered by freezing-induced morphological damage, making identification and positivity assessment difficult using traditional microscopic methods (Appendix Figure, panel B).

**Table:** *Baylisascaris procyonis* positivity rates based on necropsy and ddPCR in study of zoonotic *Baylisascaris procyonis* in raccoons, Mississippi, USA, 2023–2024*

County	Necropsy positivity rate, no. (%)	No. necropsies, N = 46	ddPCR positivity rate, no. (%)	No. ddPCR analyses
**Hancock**	0	2	**1 (50.0)**	2
Harrison	0	3	0	3
**Hinds**	0	15	**3 (17.6)**	17
**Humphreys**	**2 (50.0)**	4	**2 (50.0)**	4
**Lincoln**	0	1	**1 (100.0)**	1
**Pearl River**	0	3	**1 (33.3)**	3
**Yazoo**	**4 (57.1)**	7	**4 (57.1)**	7
Warren	0	11	0	8
**Total**	**6 (13.0)**	**46**	**12 (26.7)**	**45**

* Bold indicates Mississippi counties with raccoons positive for *B. procyonis* nematodes or for *Baylisascaris* sp. DNA. ddPCR, digital droplet PCR.

**Figure 2:**
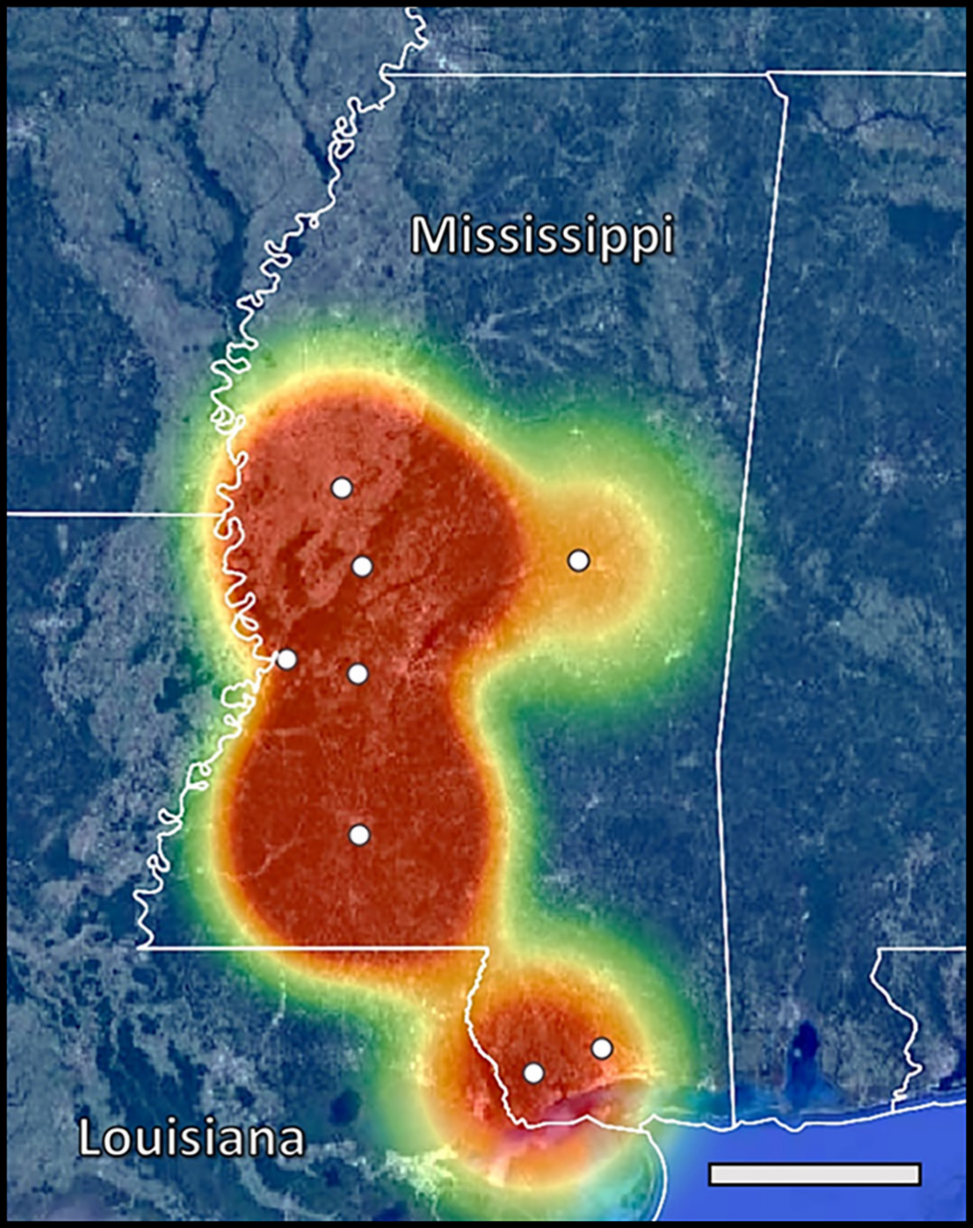
Locations of Mississippi counties sampled in study of zoonotic *Baylisascaris procyonis* in raccoons, Mississippi, USA, 2023–2024. Map indicates geographic center of counties sampled (white circles) and heatmapping estimates of *B. procyonis* positivity at the county level on the basis of fecal analysis using droplet digital PCR. Scale bar indicates 75.0 miles.

Our ddPCR analysis of fecal DNA revealed *B. procyonis* positivity in 6 of the 8 counties in Mississippi (Hancock, Hinds, Humphreys, Lincoln, Pearl River, and Yazoo) (Table). The ddPCR results estimated an infection rate of >50% in Hancock, Humphreys, Lincoln, and Yazoo Counties; the mean infection rate was 26.7% across all 8 counties surveyed. The mean + SE concentration of *Baylisascaris* sp. DNA in positive fecal samples was 2.28 + 1.84 (range 0.16–22.17) (Appendix Table 2). Heatmapping of ddPCR results estimates *B. procyonis* positivity in raccoons throughout large portions of central and southern Mississippi and high positivity along the Mississippi–Louisiana border (Figure 2).

## Conclusions

We report evidence of *B. procyonis* roundworms in Mississippi, previously reported in neighboring states in the southeastern United States, including Louisiana, Georgia, Tennessee, and Arkansas (*5*,*8*,*9*,*13*,*14*). Using necropsy, we found an overall *B. procyonis* roundworm statewide prevalence of 13.0% across all counties tested.

Fecal analysis using ddPCR detected more *B. procyonis* infections than did necropsy analyses; the average statewide positivity rate was 26.7% across all counties, similar to the 31.7% fecal prevalence reported in Louisiana (*5*) using quantitative PCR (qPCR) techniques. ddPCR analysis indicated positive results from raccoons in Handcock, Hinds, Humphreys, Lincoln, Pearl River, and Yazoo Counties. Fecal positivity using ddPCR ranged from 17.6% to 100.0%. The limitation of convenience sampling in some counties might account for higher detection rates; only 1–3 raccoons were sampled in 4 counties. Racoon infection rates also varied by season, age, racoon population crowding, and age of animals (*6*,*15*). Overall, the infection rate estimate based on ddPCR data was higher and indicated the parasite was more widespread than was suggested by visual examination through necropsy alone. This discrepancy might be explained by the difficulty of conducting comprehensive necropsy across the entire intestine of raccoons, where some nematodes might have been overlooked; the difficulty of L3 and L4 larvae detection; or the migration of intestinal nematodes into the stomach after host death. We also found that freezing fecal samples altered *B. procyonis* egg morphology, making parasite eggs harder to identify using traditional microscopic methods. Overall, fecal analysis using ddPCR proved to be a simpler, more sensitive, and more comprehensive sampling strategy, supporting it as the preferred approach to estimate infection rates for this parasite of human–health concern.

The data represented in this study are limited by the small sample size from each county, and this study serves as proof of concept that this parasite is demonstrable in Mississippi racoons, indicating the need for increased provider awareness and animal surveillance because of the human pathogenic potential of this parasite. Taken together, the data estimate low prevalence across much of the surveyed range and spots of hyperendemicity. Future large-scaled surveillance efforts to more accurately estimate infection rates and fully assess the geographic range of this parasite in Mississippi are needed. Overall, our data suggest that raccoons in Mississippi harbor *B. procyonis* infection, representing a danger to human health. 

Appendix Additional information about zoonotic *Baylisascaris procyonis* infection in raccoons, Mississippi, USA, 2023–2024
