# Supplementary material for: Zoonotic Baylisascaris procyonis Infection in Raccoons, Mississippi, USA, 2023–2024
Source: Emerg Infect Dis. 2025 Oct;31(10):2013–6. doi: 10.3201/eid3110.250658 (PMC12483112; doi:10.3201/eid3110.250658)
Supplement: Appendix — Additional information about zoonotic Baylisascaris procyonis infection in raccoons, Mississippi, USA, 2023–2024 [file 25-0658-Techapp-s1.pdf]

Article DOI: https://doi.org/10.3201/eid3110.250658

EID cannot ensure accessibility for supplementary materials supplied by authors. Readers who have difficulty accessing supplementary content should contact the authors for assistance.

Zoonotic *Baylisascaris procyonis* Infection in Raccoons, Mississippi, USA, 2023–2024

Appendix

Supplementary data:

Nematode species identification: DNA was extracted from each nematode using the PureLink Genomic DNA Kit (Invitrogen, MA, USA). Species identify was confirmed using *cox1*, *cox2*, and *18S rRNA* primers followed by Sanger sequencing (For Primers and Probes see Appendix Table 3, Eurofins Genomics, KY, USA) (*1–3*). Amplicon identity was confirmed through the NCBI BLAST search function. Sequences were submitted to Genebank under accession numbers: PV593814 (*18s*), PV594051 (*28s*), PV594407 (*ITS1*), PV600642 (*cox2*), and PV610700 - PV610709 (*cox1*)

Fecal sample analysis: DNA was extracted from fecal samples using the DNeasy PowerSoil Kit (Qiagen, Hilden, Germany), as previously described (*4*). Droplet Digital PCR (ddPCR) was chosen as the molecular detection method based on its the greater sensitivity and precision in quantifying target DNA when compared to qPCR, which can be prone to false negatives when the concentration of target DNA is low (*5–7*). DNA extracts from fecal samples and extraction negative ddH$_2$O controls were analyzed using the ddPCR platform using a species-specific ddPCR assay adapted from qPCR assays described in Gatcombe et al. (2010) and Zhao et al. (2024) (*4*,*8*), where the probe was modified to an internal TaqMan probe (Life Technologies, Carlsbad, USA) labeled with 6-FAM at the 5′ end and a minor groove binder-nonfluorescent quencher (MGBNFQ) at the 3′end. The assay reaction mixtures and cycling conditions were optimized and validated in vitro using the Bio-Rad® QX200 AutoDG Droplet Digital PCR (ddPCR) System (Instrument no. 773BR1456 and 771BR2544). To confirm the

ddPCR assay was specific for *B. procyonis* in Mississippi, the reaction and cycling conditions were cross tested, in triplicate reactions, using 0.20 ng gDNA from the target species and three representative exclusion species, *Macracanthorhynchus ingens, Physaloptera sibirica,* and *Hysterothylacium tetrapter*. Optimized ddPCR reaction mixtures contained 1.1 µL of extracted DNA, 1X Bio-Rad® ddPCR supermix for probes (no deoxyuridine triphosphate (dUTP)), 1200 nM of each primer, and 500 nM of probe, adjusted to 22 µL using PCR-grade water, as per the manufacturer protocol for automated droplet generation (Bio-Rad® Laboratories 2014, CA, USA). Using an automated droplet generator, 20 µL of each ddPCR reaction mixture was combined with ~70 µL of automated droplet generation oil to create ~20,000 nanoliter-sized droplets before PCR cycling (Bio-Rad® Laboratories 2014). Optimal ddPCR cycling conditions were enzyme activation at 95°C for 10 min, followed by 40 cycles of: 94°C for 30 s and 60°C for 2 min, with a final enzyme deactivation step at 98°C for 10 min, using a ramp rate of 2°C/s. These ddPCR reaction and cycling conditions successfully amplified DNA from all replicates of *B. procyonis*, but not from any replicates for the exclusion species. All fecal DNA extracts were run on the ddPCR platform in triplicate reactions. Negative controls were included on each plate, also conducted in triplicate. To reduce the likelihood of contamination across sample processing stages, ddPCR reactions were prepared and run in a separate laboratory from those where raccoon necropsies and DNA extractions took place. Additional precautions were also implemented to reduce the risk of contamination and ensure authenticity of ddPCR data, following recommendations by Lehman et al. (2020) for environmental DNA studies (*5*). All ddPCR replicates for the DNA extraction and ddPCR negative controls met none of the criteria for positive detection, indicating samples were not contaminated during lab processing. Eggs from ddPCR-confirmed fecal samples were floated in a 33% zinc sulfate solution and imaged on a coverslip with an Olympus Tokyo invert microscope.

ddPCR analysis and QGIS mapping: Samples and negative controls were analyzed using a three criteria approach to reduce the likelihood of false positives using previously described methods (*5*,*6*). To be defined as a positive detection of *Baylisascaris* sp. DNA, all three criteria had to be met. For negative controls to be deemed free from contamination, none of the data analysis criteria could be met. The three analysis criteria were: 1) droplets were above the MT, 2), droplets fell within the known positive droplet range for the assay, and 3) the concentration of target DNA was greater than or equal to the Limit of Detection (LOD) for the assay. The MT for

the assay was defined by analyzing 48 ddPCR no template control (NTC) reactions, containing no target DNA. The highest amplitude of the NTC reactions was ≈1500 RFUs above the negative band, therefore the MT was conservatively set to be 3000 RFUs above the negative band to reduce the incidence of false positives. The positive droplet range was defined during in vitro validation as 5,500 – 7,000 RFUs above the negative band, at 8,000 – 9,500 RFUs during The LOD of the assay was defined by screening a 7-fold series of 10X dilutions from starting concentrations of 2.0 ng/µL X 10^{-8} gBlock Gene Fragments for the target locus, in triplicate reactions, quantified using BioRad QuantaSoft Rare Event Detection (RED) analysis. Target DNA was detectable in all replicate dilutions up to 2.0 X 10^{-8}, not in the 2.0 X 10^{-9} dilutions. A subsequent 2X dilution series was run, in triplicate, from the 2.0 X 10^{-8} dilution to further refine LoD. DNA was detected the 1.0 X 10^{-8} dilution, and the standard errors for these dilutions did not include zero, nor did they overlap with the standard errors of the 5.0 X 10^{-9} dilutions. Using the average number of copies of target DNA/µL in the 1.0 X 10^{-8} dilution and applying the lower standard error as the relaxed detection threshold, the LOD of the assay was determined to be 0.06 copies/µL using methods previously described (*7*). QGIS software and ddPCR positivity data were used to create a heatmap of Mississippi based upon the geographic center of each county surveyed using methods as previously described (*9*).

Phylogenetic analysis: Phylogenetic analyses were conducted using both maximum likelihood (MEGA 11) and Bayesian inference (MrBayes) methods, employing the GTR + gamma model. These analyses were based on partial sequences of *COX1* gene (418bp) from isolates of *Baylisascaris* spp. *Toxascaris lenonina* was used as an outgroup. Bayesian posterior probabilities and bootstrap support values (1000 bootstrap replicates) were calculated for each node.

References

1. Franssen F, Xie K, Sprong H, van der Giessen J. Molecular analysis of Baylisascaris columnaris revealed mitochondrial and nuclear polymorphisms. Parasit Vectors. 2013;6:124. PubMed https://doi.org/10.1186/1756-3305-6-124

2. Kounosu A, Murase K, Yoshida A, Maruyama H, Kikuchi T. Improved 18S and 28S rDNA primer sets for NGS-based parasite detection. Sci Rep. 2019;9:15789. PubMed https://doi.org/10.1038/s41598-019-52422-z

3. Gu XH, Chen HX, Hu JJ, Li L. Morphology and ASAP analysis of the important zoonotic nematode parasite *Baylisascaris procyonis* (Stefahski and Zarnowski, 1951), with molecular phylogenetic relationships of *Baylisascaris* species (Nematoda: Ascaridida). Parasitology. 2024;151:200–12. PubMed

4. Zhao H, Zendejas-Heredia PA, Colella V, Arguello I, Brookes K, Williams JM, et al. Surveillance of soil-transmitted helminths and other intestinal parasites in shelter dogs, Mississippi, USA. One Health. 2024;20:100956. **PMID 39834887**

5. Lehman RN, Poulakis GR, Scharer RM, Schweiss KE, Hendon JM, Phillips NM. An environmental DNA tool for monitoring the status of the Critically Endangered Smalltooth Sawfish, Pristis pectinata, in the western Atlantic. Conserv Genet Resour. 2020;12:621–9. https://doi.org/10.1007/s12686-020-01149-5

6. Schweiss KE, Lehman RN, Drymon JM, Phillips NM. Development of highly sensitive environmental DNA methods for the detection of Bull Sharks, Carcharhinus leucas (Müller and Henle, 1839), using Droplet Digital™ PCR. Environ DNA. 2020;2:3–12. https://doi.org/10.1002/edn3.39

7. Baker CS, Steel D, Nieukirk S, Klinck H. Environmental DNA (eDNA) from the wake of the whales: Droplet digital PCR for detection and species identification. Front Mar Sci. 2018;5:133. https://doi.org/10.3389/fmars.2018.00133

8. Gatcombe RR, Jothikumar N, Dangoudoubiyam S, Kazacos KR, Hill VR. Evaluation of a molecular beacon real-time PCR assay for detection of *Baylisascaris procyonis* in different soil types and water samples. Parasitol Res. 2010;106:499–504. PubMed https://doi.org/10.1007/s00436-009-1692-6

9. Hearst S, Huang M, Johnson B, Rummells E. Identifying Potential Super-Spreaders and Disease Transmission Hotspots Using White-Tailed Deer Scraping Networks. Animals (Basel). 2023;13:1171. PubMed https://doi.org/10.3390/ani13071171

Appendix Table 1. Mean (±STDEV) number and length of *Baylisascaris procyonis* nematodes found in visually infected adult and juvenile raccoons.

Age	Number	Length (cm)
Adults	5.3 ± 2.8	11.1 ± 2.4
Juvenile	41.5 ± 7.7	6.2 ± 0.8

Appendix Table 2. Mean±SE concentrations (copies/μL) of *Baylisascaris sp*. DNA from positive fecal samples measured using Droplet Digital PCR Rare Event Detection.

ID	Mississippi County	Mean±SE copies/μL
S2	Hancock	0.34 ± 0.05
H4	Hinds	0.53 ± 0.12
H10	Hinds	0.45 ± 0.13
R3	Hinds	0.16 ± 0.03
HU2	Humphreys	0.91 ± 0.05
HU4	Humphreys	22.17 ± 0.11
L1	Lincoln	0.22 ± 0.07
S8	Pearl River	0.44 ± 0.09
B1	Yazoo	0.73 ± 0.20
B3	Yazoo	0.20 ± 0.04
B5	Yazoo	0.34 ± 0.05
B6	Yazoo	0.84 ± 0.20

Appendix Table 3. Primers and Probes for Sanger Sequencing and Droplet Digital PCR.

Name	Sequence	Gene
Bpro-F	5′-GAGTTATGAGTTTAGTGATATTCCTGGA-3′	*cox2*
Bpro-R	5′-GCAAAGCCCAAGAATGAATCAC-3′	
Bpro-Pr	5′-6FAM-TATTAACATCACAAGGTACAACACAACG-MGBNFQ-3′	
CO1-F	5′-TTTTTTGGGCATCCTGAGGTTTAT-3′	*cox1*
CO1-R	5′-TAACGACATAACATAATGAAAATG-3′	
BPC2-F	5′-GAGTTATGAGTTTAGTGATATTCCTGGA-3′	*cox2*
BPC2-R	5′-GCAAAGCCCAAGAATGAATCAC-3′	
B28-F	5′-CGAGGATTCCCTTAGTAACT-3′	*28s*
B28-R	5′-TCGGATAGGTGGTCAACG-3′	
563-F	5′-GCCAGCAVCYGCGGTAAY-3′	*18s*
1132-R	5′-CCGTCAATTHCTTYAART-3′	
BIT1F	5′-ATAGTGAGTTGCACACTAATGT-3′	*ITS1*
BIT1R	5′-TTATATGCTTAAATTCAGCGGG-3′	

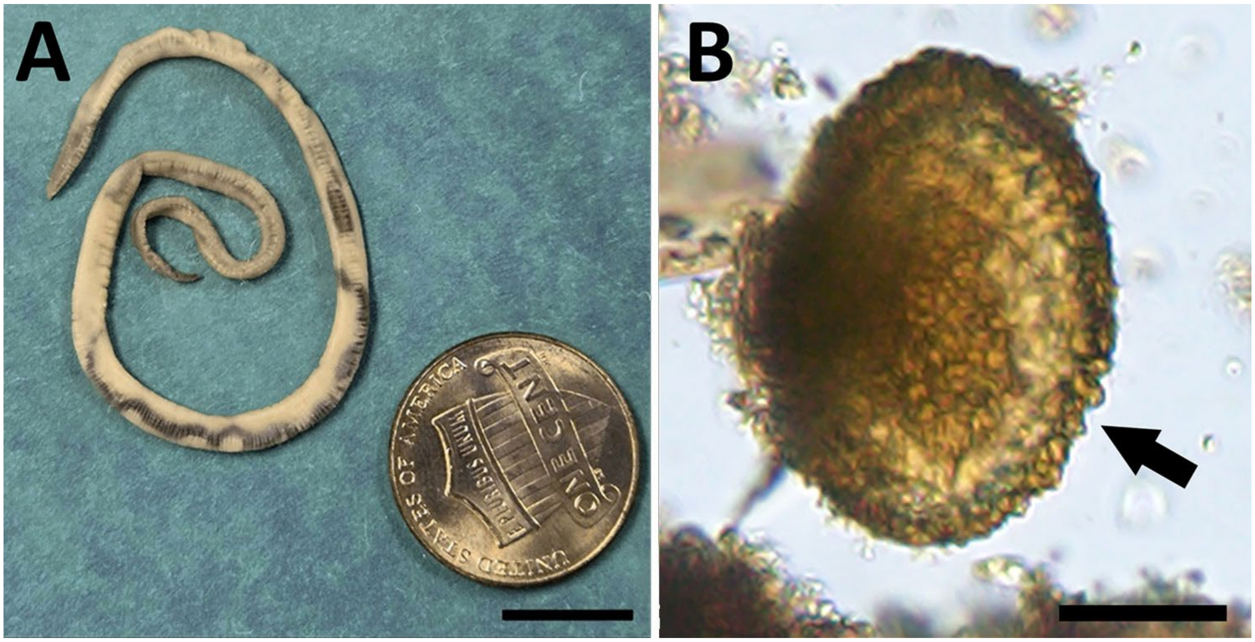

Appendix Figure. (A) Adult *Baylisascaris procyonis* nematode collected by necropsy from a raccoon in Mississippi. United States penny used for scale (scale bar: 1.0 cm). (B) *Baylisascaris procyonis* egg from Droplet Digital PCR positive fecal sample. Note arrow pointing to altered membrane from −20°C freezer storage. (scale bar: 35.0 μm).
